# Supplementary material for: Identification of drug candidates that enhance pyrazinamide activity from a clinical compound library
Source: Emerg Microbes Infect. 2017 Apr 26;6(4):e27–. doi: 10.1038/emi.2017.23 (PMC5457674; doi:10.1038/emi.2017.23)
Supplement: Supplementary Table S1 [file emi201723x1.pdf]

Supplementary Table S1 Activity of 130 clinical compounds that are active against a 3-month-old *M. tuberculosis* H37Ra culture in combination with PZA (100 μg/ml)

**Drugs**	**FDA/Indication**	**Viability of bacteria after 3, 5, or 7 days of drug exposure [a]**		
		3d	5d	7d
Negative control		+	+	+
PZA	TB drug	+	+	+
**Gold Au 79**	**FDA**/Antineoplastic	— [b]	—	—
Quinaldine blue	**FDA**/Antineoplastic	—	—	—
Carboplatin	**FDA**/Antineoplastic	—	—	—
**Verteporfin**	**FDA**/Antineoplastic	—	—	—
Decitabine	**FDA**/Antineoplastic	—	—	—
**Clotrimazole**	**FDA**/Antifungal	—	—	—
Sulconazole	**FDA**/Antifungal	—	—	—
Bifonazole	Antifungal	—	—	—
Conazole	Antifungal	—	—	—
Tioconazole	**FDA**/Antifungal	—	—	—
Butoconazole	**FDA**/Antifungal	—	—	—
Miconazole	**FDA**/Antifungal	—	—	—
Oxiconazole	Antifungal	—	—	—
Ciclopirox ethanolamine	**FDA**/Antifungal	+ [c]	—	—
**Doxycycline**	**FDA**/Antibiotic	—	—	—
Minocycline	**FDA**/Antibiotic	—	—	—
Levofloxacin	**FDA**/Antibiotic	+	—	—
Moxifloxacin	**FDA**/Antibiotic	+	+	—
Amikacin	**FDA**/Antibiotic	—	—	—
Gatifloxacin	**FDA**/Antibiotic	—	—	—
Tetracycline	**FDA**/Antibiotic	—	—	—
Nifuroxazide	Antibiotic	—	—	—
Chlortetracycline	**FDA**/Antibiotic	—	—	—
Enrofloxacin	Antibiotic	+/— [d]	+/—	—
**Fleroxacin**	Antibiotic	+/—	+/—	—
**Florfenicol**	Antibiotic	+/—	+/—	—
**Nitroxoline**	Antibiotic	—	—	—
**Rifamycin**	**FDA**/Antibiotic	—	—	—
**Thiostrepton**	Antibiotic	—	—	—
**Tosufloxacin**	Antibiotic	+/—	+/—	—
Monensin sodium	Antibiotic	—	—	—
Streptomycin	**FDA**/Antibiotic	+	+/—	—
Isoniazid	**FDA**/Antibiotic	+/—	+/—	—

**Nifuroxime**	**FDA**/Antibiotic	+	+/−	−
Dapsone	**FDA**/Antibiotic	−	−	−
Formylrifamycin	Antibacterial	−	−	−
Aminosalicylic acid	**FDA**/Antibacterial	+	+/−	−
Carbadox	Antibacterial	−	−	−
**Cloxyquin**	Antibacterial	−	−	−
Diclazuril	Antibacterial	−	−	−
Arsanilic acid	Antibacterial	+	+/−	−
Betamipron	Antibacterial	−	−	−
**Clopidol**	Antibacterial	−	−	−
Meclocycline	**FDA**/Antibacterial	−	−	−
Merbromin	**FDA**/Antibacterial	−	−	−
**Pyrithione zinc**	**FDA**/Antibacterial	−	−	−
**Silver Sulfadiazine 1%**	**FDA**/Antibacterial	+/−	−	−
Atovaquone	**FDA**/Antimalarial	−	−	−
**Clioquinol**	**FDA**/Antibacterial	−	−	−
Domiphen bromide	**FDA**/Antibacterial	−	−	−
Silver nitrate	**FDA**/Antibacterial	−	−	−
Iodoform	**FDA**/Antibacterial	−	−	−
**Clofazimine**	**FDA** /Antibacterial	−	−	−
Bismuth tribromophenate	**FDA**/Antibacterial	+/−	−	−
Chloramine-T hydrate	**FDA**/Antibacterial	+/−	−	−
Chlorquinaldol	**FDA**/Antibacterial	+	+	−
Povidone iodine	**FDA**/Antibacterial Antiseptic	−	−	−
Benzalkonium chloride	**FDA**/Antiseptic	−	−	−
Bithionol	**FDA**/Antiseptic	−	−	−
Gentian violet	**FDA**/Antiseptic	−	−	−
Cetylpyridinium	**FDA**/Antiseptic	−	−	−
Iodine	**FDA**/Antiseptic, Thyroid	−	−	−
Crospovidone	**FDA**/Antibacterial Antiseptic	−	−	−
**Thonzonium Bromide**	**FDA** /Antiseptic	−	−	−
Methylbenzalkonium chloride	**FDA**/Antiseptic	−	−	−
Dibromosalicylaldehyde	Antiseptic	−	−	−
Triclocarban	**FDA**/Antiseptic	−	−	−
Veratrole	**FDA**/Antiseptic	+	−	−
Ricobendazole	Anthelminithic	+	+/−	−
Bitoscanate	Anthelminithic	−	−	−
**Oxantel**	Anthelminithic	−	−	−
Tetramisole	Anthelminithic	−	−	−
Tetrachloroethylene	Anthelminithic	−	−	−
**Closantel**	**FDA**/Anthelminithic	−	−	−

Bephenium hydroxynaphthoate	**FDA**/Anthelminithic	—	—	—
Dithiazanine iodide	**FDA**/Anthelminithic	—	—	—
Pyrvinium pamoate	**FDA**/Anthelminithic	—	—	—
Meloxicam	**FDA**/Antiinflammatory	—	—	—
Meclofenamic Acid	**FDA**/Antiinflammatory	—	—	—
Tolfenamic acid	Antiinflammatory	—	—	—
Acemetacin	Antiinflammatory	—	—	—
Flufenamic acid	Antiinflammatory	—	—	—
Niflumic acid	Antiinflammatory	—	—	—
Indomethacin	**FDA**/Antiinflammatory	—	—	—
Chlorosalicylanilide 0.5%	**FDA**/Antiinflammatory	—	—	—
Vitamin K5	**FDA**/Vitamin	—	—	—
Vitamin D3	**FDA**/Vitamin	—	—	—
Vitamin D2	**FDA**/Vitamin	—	—	—
Irbesartan	**FDA**/Antihypertensive	+	+/—	—
Candesartan cilexetil	**FDA**/Antihypertensive.,	—	—	—
Fluvastatin sodium	**FDA**/Antihyperlipidemic	—	—	—
Glimepiride	/Antidiabetic	+	—	—
Clemizole hydrochloride	**FDA**/Antihistaminic	—	—	—
Octodrine	**FDA**/Decongestant	—	—	—
Nifedipine	**FDA**/Antianginal	—	—	—
Amiodarone hydrochloride	**FDA**/Antiarrhythmic	—	—	—
Tolonium chloride	**FDA**/Antihypertensive	—	—	—
Cholic acid	**FDA**/Choleretic	—	—	—
Danthron	**FDA**/Laxative	—	—	—
Deoxycholic acid	**FDA**/Choleretic	—	—	—
**Rose bengal**	**FDA**/Diagnostic aid	—	—	—
Iopanoic acid	**FDA**/Diagnostic aid	—	—	—
Hydroxyzine	**FDA**/Anxiolytic	—	—	—
Ferrous sulfate	**FDA**/Hematinic	—	—	—
Polidocanol	Anesthetic	—	—	—
Captan 0.1%W/V	**FDA**/Phamaceutic aid	—	—	—
Mepirizole	Analgesic	+	—	—
Thyropropic acid	Antihyperlipidemic	—	—	—
Benzbromarone	Uricosuric	—	—	—
**Gold sodium thiomalate**	**FDA**/Antirheumatic	—	—	—
Ditiocarb	**FDA**/Immunomodulator	—	—	—
Myristic acid	Pharmaceutic aid	+	+	—
Lansoprazole	**FDA**/Antiulcerative	—	—	—
Omeprazole	**FDA**/Antiulcerative	—	—	—
Suloctidil	Vasodilator	—	—	—
Homidium bromide	Anthelminithic	—	—	—
Carbenoxolone	Antiulcerative	—	—	—

Primaquine	**FDA/**Antimalarial	＋	＋/－	－
Ricinoleate	Unclassified	－	－	－
Carzenide	Unclassified	＋	＋	－
Ethoxyquin	Unclassified	－	－	－
Fusaric acid	Unclassified	－	－	－
**Cod liver oil**	Unclassified	－	－	－
Olive oil	Unclassified	－	－	－
Thymol	**FDA/**Antibacterial	－	－	－
Storax	Unclassified	－	－	－

[a] A 3-month-old *M. tuberculosis* H37Ra culture was treated with different compounds (50 µM) and PZA (100 µg/ml) for 3, 5, or 7 days, when the viability of the bacteria was determined by transfer to 7H11 plates using a 96-pin replicator after compound exposure.

[b] －: No colonies grew on 7H11 plates.

[c] ＋: Obvious colonies grew on 7H11 plates.

[d] ＋/－: Small faint colonies grew on 7H11 plates.
